# Monitoring and Inhibiting MT1-MMP during Cancer Initiation and Progression

**DOI:** 10.3390/cancers6010416

**Published:** 2014-02-17

**Authors:** Sonia Pahwa, Maciej J. Stawikowski, Gregg B. Fields

**Affiliations:** 1 Department of Pharmaceutical Sciences, College of Pharmacy, The University of Oklahoma, 1110 North Stonewall Avenue, Oklahoma City, OK 73117, USA; E-Mail: pahwa.sonia@gmail.com; 2 Departments of Chemistry and Biology, Torrey Pines Institute for Molecular Studies, Port St. Lucie, FL 34987, USA; E-Mail: mstawikowski@tpims.org

**Keywords:** MT1-MMP, MMP-14, matrix metalloproteinases, CD44, collagen, metastasis

## Abstract

Membrane-type 1 matrix metalloproteinase (MT1-MMP) is a zinc-dependent type-I transmembrane metalloproteinase involved in pericellular proteolysis, migration and invasion. Numerous substrates and binding partners have been identified for MT1-MMP, and its role in collagenolysis appears crucial for tumor invasion. However, development of MT1-MMP inhibitors must consider the substantial functions of MT1-MMP in normal physiology and disease prevention. The present review examines the plethora of MT1-MMP activities, how these activities relate to cancer initiation and progression, and how they can be monitored in real time. Examination of MT1-MMP activities and cell surface behaviors can set the stage for the development of unique, selective MT1-MMP inhibitors.

## 1. Introduction

**Figure 1:**
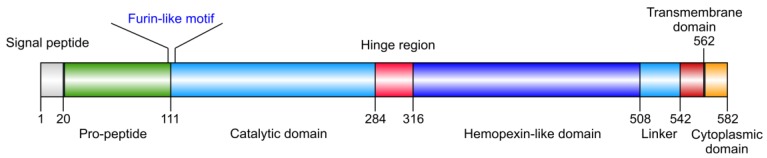
Structural domains of MT1-MMP. The catalytic (CAT) and hemopexin-like (HPX) domains are common to most MMPs, while the transmembrane (TM) and cytoplasmic (CT) domains are unique to MT-MMPs.

**Figure 2:**
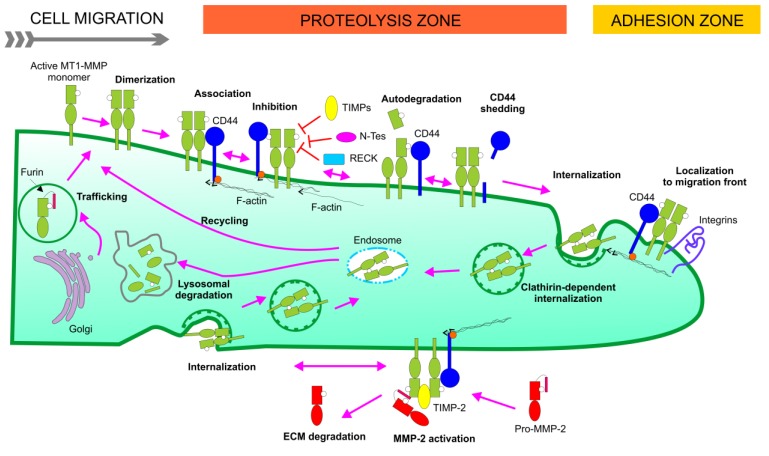
Schematic presentation of MT1-MMP activation, activity, and disappearance at the cell surface. Derived from prior overviews of MT1-MMP at the cell surface [6,7].

Membrane-type 1 matrix metalloproteinase (MT1-MMP) is widely expressed and its presence has been documented in multiple cell types. MT1-MMP is distinguished from soluble MMPs by *C*-terminal transmembrane and cytoplasmic domains (Figure 1). MT1-MMP is regulated at the transcriptional and post-transcriptional levels both as a proteinase and as a membrane protein. Regulation of MT1-MMP includes: (1) expression/mRNA stabilization/protein production, (2) activation by furin, (3) subcellular localization, (4) internalization, (5) recycling, (6) dimerization, (7) molecular associations, (8) shedding, (9) posttranslational modifications (glycosylation, phosphorylation, palmitoylation, disulfide bond formation), and (10) endogenous inhibitors [tissue inhibitor of metalloproteinase (TIMP), reversion-inducing-cysteine-rich protein with kazal motifs (RECK)] (Figure 2) [1,2,3,4]. MT1-MMP is produced as an inactive ∼60 kDa zymogen that is activated by furin-like convertases, which cleave at the Arg108-Arg-Lys-Arg motif located between the propeptide and the catalytic (CAT) domain. Active MT1-MMP (∼57 kDa), starting at Tyr112, is then transported to the plasma membrane with the CAT domain facing the extracellular space, where it cleaves pericellular substrates. Active MT1-MMP undergoes autocatalytic processing at the cell surface, leading to the formation of an inactive 44 kDa fragment and release of the entire CAT domain. First, MT1-MMP cleaves itself at the Gly284-Gly285 peptide bond in the hinge region, generating the inactive 44 kDa membrane-bound fragment. The second cleavage takes place at the Ala255-Ile256 bond, in the active site of MT1-MMP. The released 18 kDa soluble fragment has no catalytic activity and does not bind TIMP-2 [5]. The autocatalytic cleavage is thus a self-regulatory mechanism that terminates MT1-MMP-dependent proteolysis both at the cell surface and in the extracellular space.

## 2. Implication in Cancer

MT1-MMP is found overexpressed in many tumors [8]. The expression of MT1-MMP is associated with poor prognosis in patients with advanced neuroblastoma, small cell lung cancer, tongue squamous cell carcinoma, head and neck carcinoma, bladder cancer, breast cancer, and ovarian cancer [9,10]. Increased tumor cell expression of MT1-MMP enhances tumor growth, invasion, and metastasis [11]. MT1-MMP induces the epithelial to mesenchymal transition in prostate and squamous cell carcinoma cells [12,13]. In pancreatic ductal adenocarcinoma, MT1-MMP processing of collagen and/or activation of growth factors enhances signaling pathways that eventually promote chemoresistance [14]. MT1-MMP is generally considered pro-invasive and pro-tumorigenic as: (a) the expression and the activity of MT1-MMP are elevated in tumor cells and (b) high levels of MT1-MMP directly correlate with enhanced cell migration [15].

Degradation of the extracellular matrix (ECM) by MT1-MMP has been linked to proliferation, invasion, and metastasis of cancer cells in most cancer types. Gene mutations have not been observed for MT1-MMP in cancer, therefore up-regulation of MT1-MMP has been linked to the transcriptional changes during tumor formation. Immunohistochemical and *in-situ* hybridization analysis have demonstrated the presence of MT1-MMP in both tumor cells and stromal cells [16]. Interestingly, MT1-MMP can be produced by the stromal cells rather than the cancer cells in several tumor types. It is possible that extracellular matrix metalloproteinase inducer (EMMPRIN) is produced on the cell surface of cancer cells, which in turn stimulates the surrounding stromal cells to produce MMPs [17]. Conversely, the majority of the MT1-MMP expression in thyroid, brain, and head and neck cancer is from the cancer, rather than the surrounding stromal, cells [16].

MT1-MMP has also been shown to regulate transcriptional programs in a number of cell lines [18]. Overexpression of MT1-MMP in the breast cancer cell line MCF-7 increased transcription of vascular endothelial growth factor A (VEGF-A) and, concurrently, tumor growth, angiogenesis, and metastasis [19]. Transcription of VEGF-A was regulated through MT1-MMP catalytic activity and the cytoplasmic domain, as well as Src kinase activity. Overexpression of MT1-MMP also increased transcription of the gene encoding VEGF-A and tumor growth in U251 cells [20]. Transcription of dickkopf-related protein 3 (DKK3) in urothelial cells and Smad1 in several tumor cell lines was regulated by MT1-MMP [18]. 

## 3. MT1-MMP Substrates

The activity of MT1-MMP was initially reported as a membrane associated promatrix metalloproteinase-2 (proMMP-2) activator [21]. ProMMP-2 activation involves a trimolecular complex consisting of MT1-MMP, proMMP-2, and TIMP-2. Although TIMP-2 is an inhibitor of MMPs, low concentrations of TIMP-2 aid in the activation of proMMP-2 by MT1-MMP within this complex by binding to the CAT domain of MT1-MMP and the *C*-terminal domain of proMMP-2. Once the MT1-MMP•TIMP-2•proMMP-2 complex is formed, a second MT1-MMP forms a dimer with the MT1-MMP in the complex and cleaves proMMP-2 to produce active MMP-2. Active MMP-2 can then process ECM proteins not cleaved by MT1-MMP. 

MT1-MMP also activates proMMP-13. Stromal expression of MMP-13 is a critical factor in melanoma invasion and metastasis [22]. In similar fashion, breast cancer cells induce MMP-13 production from osteoblasts, and the subsequent MMP-13 action on a variety of substrates promotes metastasis to the bone [23]. A selective MMP-13 inhibitor was found to delay breast cancer growth and cancer-induced bone osteolysis [24]. MMP-13 promotes head and neck cancer angiogenesis [25].

Several proteomics approaches have been used to identify MT1-MMP substrates. MT1-MMP CAT was treated with plasma proteins and the products identified by two-dimensional gel electrophoresis and mass spectrometry (MS) [26]. A total of 15 different protein substrates were identified, including fibrinogen, vitronectin, apolipoprotein A-I, apolipoprotein E, and plasma gelsolin, and validated *in vitro* [26].

The quantitative MS based proteomic technique isotope-coded affinity tag (ICAT) labeling was used in a cell-based substrate discovery screen of MT1-MMP and led to the identification of 14 novel MT1-MMP candidate substrates, only two of which were ECM proteins [27]. The remaining proteins encompassed cytokines, chemokines, cell receptors and serine proteinase inhibitors. A similar approach was used to examine the effect of the MMP inhibitor prinomastat on MT1-MMP expressing MDA-MB-231 cells [28]. The membrane and secreted proteomes of cells were analyzed, providing insight into the effect of both MT1-MMP and prinomastat on cell membrane ectodomain shedding. Over 25 known MMP and MT1-MMP substrates were identified, validating the technique, as well as over 40 novel substrates with diverse functions, twenty of which were biochemically validated *in vitro* in the same study (including DJ-1, galectin-1, Hsp90alpha, pentraxin 3, progranulin, Cyr61, peptidyl-prolyl *cis*-*trans* isomerase A, and dickkopf-1).

MT1-MMP hydrolyzes ECM and serum proteins (type I collagen, fibronectin, vitronectin, laminin-1, the laminin-5 γ2 chain, apolipoproteins, fibrillar amyloid β-protein, fibrinogens, and vitronectin), sheds cell surface biomolecules [CD44, syndecan-1, death receptor-6, MHC class I chain-related molecule A, E-cadherin, low density lipoprotein receptor-related protein 1 (LRP1/CD91), mucin 1, tissue *trans*-glutaminase, and extracellular matrix metalloproteinase inducer (EMMPRIN)] and cytokines, chemokines, and growth factors (neutrophil chemokine IL-8, secretory leukocyte protease inhibitor, pro-tumor necrosis factor, death receptor-6, connective tissue growth factor, SDF-1 and TGF-β), and activates the pro-αv integrin subunit [3,13,17,20,21,26,27,28,29,30,31,32,33,34,35,36]. Intracellular substrates of MT1-MMP, such as pericentrin and breast cancer type 2 susceptibility gene (BRCA2), have also been identified [37,38]. The regulation of a variety of cellular functions, such as motility, invasion, growth, differentiation, and apoptosis, is altered by MT1-MMP processing of the above substrates. 

MT1-MMP stimulates epithelial cell, fibroblast, and cancer cell invasion into collagen, while the other collagenases (MMP-1, MMP-2, MMP-8, and MMP-13) do not [39,40]. In general, MT1-MMP increases cell migration and invasion by degrading components of the ECM and making a path through surrounding tissue. Facilitating this process is the concentration of MT1-MMP in the protrusions of cells (lamellipodia in normal cells and invadopodia in tumor cells). ECM catabolism by MT1-MMP promotes focal adhesion turnover and ERK activation, reduces FAK phosphorylation, and ultimately stimulates cell migration [34]. MT1-MMP cleavage of E-cadherin, a cell adhesion molecule, disrupts contact between cells.

EMMPRIN is cleaved by MT1-MMP between the two extracellular Ig-like domains. The shed fragment can stimulate fibroblasts to express MMPs. Thus, MT1-MMP shedding of EMMPRIN could mediate MMP induction in the tumor stroma [17].

Active MT1-MMP cleaves LRP1. However, if MT1-MMP is inhibited by TIMP-2, the MT1-MMP•TIMP-2 complex binds, but does not cleave, the LRP1 receptor. It is possible that as a result of this binding event, LRP1 signals through its heavily phosphorylated cytoplasmic tail [13]. LRP1 signaling is well known to stimulate the ERK/MEK pathway. The MT1-MMP•TIMP-2•LRP1 complex is then internalized by the cells causing a decay of the signal [13].

The discoidin domain receptors (DDRs) are receptor tyrosine kinases that bind to collagens. Collagen-dependent DDR1 activation is partly regulated by the proteolytic activity of MT1-MMP, which cleaves DDR1 [41]. Constitutive shedding of endogenous DDR1 in breast cancer HCC1806 cells is partly mediated by MT1-MMP, which also regulates collagen-induced receptor activation [41].

## 4. Non-Catalytic Role for MT1-MMP

Binding of MT1-MMP by low, physiological levels of TIMP-2 result in induction of the Ras-Raf-ERK signaling cascade, ultimately promoting tumor cell migration [13,42]. Signaling induction and cell migration can occur with the catalytically-inactive enzyme, as pathway induction is based on TIMP-2 binding to the HPX domain of MT1-MMP [13,42]. Growth of tumor xenografts expressing wild type or catalytically inactive MT1-MMP greatly exceeded that of tumors that expressed no MT1-MMP [13,42].

During mammary gland branching morphogenesis, mammary epithelial cells (MECs) invade the surrounding stroma. The catalytic activity of MT1-MMP is required for MEC branching in dense but not sparse three-dimensional collagen gels, while a non-proteolytic function of MT1-MMP is required for branching in both conditions [43]. Silencing MT1-MMP reduces the levels of the β1 integrin subunit (Itgb1), which is required for branching *in vivo*, and MT1-MMP associates directly with Itgb1 through the transmembrane domain/cytoplasmic tail in MT1-MMP [43]. Notably, this non-catalytic domain is required for branching in collagen gels and thus the nonproteolytic activities of MT1-MMP modulate the Itgb1-dependent signals that mediate MEC invasion during branching morphogenesis [43]. The above studies provide a possible explanation for why cancer therapy drugs that target the catalytic function of MT1-MMP may have failed in clinical trials.

## 5. MT1-MMP Binding Partners

**Figure 3:**
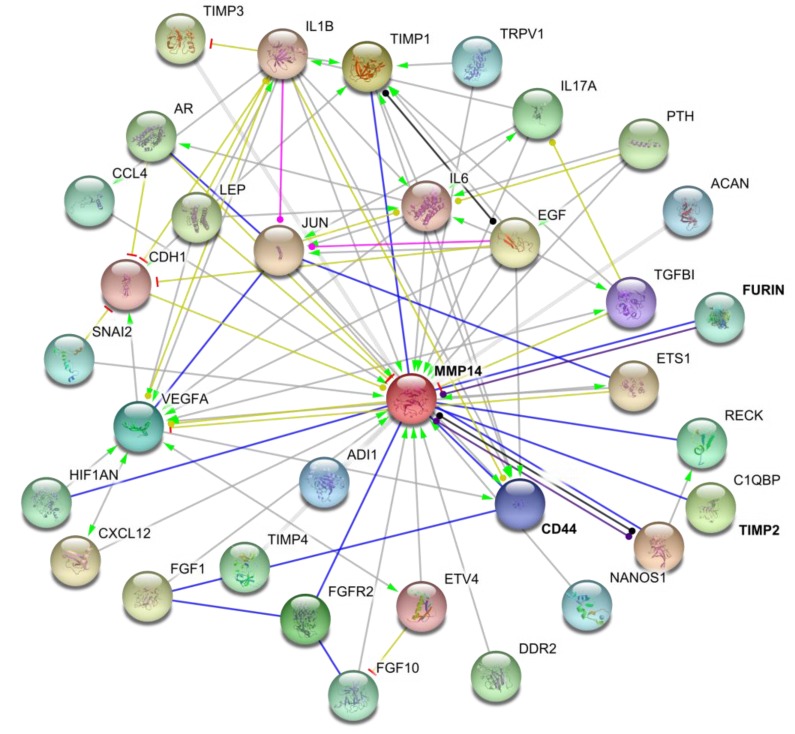
Interactions of MT1-MMP (MMP-14) with different cellular partners. Interaction map generated using STRING database [44].

Mass spectrometic analysis of biotin-labeled cell surface proteins revealed 158 binding partners for MT1-MMP [9]. Numerous MT1-MMP cell surface binding partners have been validated [44], including tetraspanins (CD9, CD37, CD53, CD63, CD81, CD82, CD151, and/or TSPAN12), the α2β1 and αvβ3 integrins, and CD44 (Figure 3) [6,9,45,46,47,48,49,50]. The interaction with tetraspanins is part of a ternary complex that includes the α3β1 integrin [6,47]. Tetraspanins protect newly synthesized MT1-MMP from lysosomal degradation and support delivery to the cell surface [6,50]. The HPX domain of MT1-MMP binds to CD63 and CD151 [47,51]. MT1-MMP association with CD151 in endothelial cells modulates collagenolysis, in that knockdown of CD151 decreases collagenolysis [47]. Conversely, co-transfection of COS cells with MT1-MMP and either CD9, CD37, CD63, CD81, or CD82 decreases invasion of collagen gels [50]. Knockdown of relevant tetraspanins has been reported to both decrease and increase MT1-MMP proteolytic activities [6,47]. Affected functions include fibronectin proteolysis, invasion and growth in three-dimensional fibrin and collagen gels, and proMMP-2 activation. The effects of tetraspanins on MT1-MMP activity may be dependent on the specific tetraspanin and/or cell type [50]. MT1-MMP does not seem to catalyze tetraspanin proteolysis.

CD44 binds to MT1-MMP via blade I of the HPX domain [8,46]. The association with CD44 leads to MT1-MMP localization to lamellipodia [46,51]. The interaction of MT1-MMP with CD44 promotes signaling through EGFR activation to the MAPK and PI3K pathways, enhancing cell migration [8]. Both MT1-MMP and ADAM-10 have been implicated for constitutive shedding of CD44 from the human melanoma cell surface [52,53]. However, the full spectrum of proteases that may participate in CD44 shedding has yet to be defined, as MMP-9 and a chymotrypsin-like enzyme have also been described as CD44 sheddases [54,55]. 

In order to examine CD44 shedding, our laboratory designed, synthesized, and characterized a series of peptides and glycopeptides spanning the stem region of CD44 (Table 1). The peptides were examined as potential substrates for MT1-MMP, MMP-2, MMP-8, MMP-9, ADAM-10, and ADAM-17. None of the peptides tested was digested by any of the enzymes. It has been reported that the CD44-CS1 peptide was cleaved *in vitro* by MT1-MMP, while shorter CD44-derived peptides were not [52]. Under our experimental conditions this peptide was not hydrolyzed by full-length MT1-MMP. Conversely, intact CD44 was hydrolyzed by full-length MT1-MMP, suggesting that the conformation of the substrate is an important contributor to its proteolysis. Using mice carrying null mutations for CD44 or the β3 integrin subunit, Chun *et al*. demonstrated that MT1-MMP collagenolytic and tubulogenic activities were not significantly affected [56]. Alternatively, the co-expression of MT1-MMP and the β3 integrin subunit, compared with MT1-MMP alone, conferred an invasive phenotype on MCF-7 cells [20]. It is possible that the binding partners are not needed for absolute MT1-MMP activity, but rather serve to modulate MT1-MMP activity or other downstream MT1-MMP functions. 

**Table 1:** CD44 sequence and (glyco)peptides derived from the stem region.

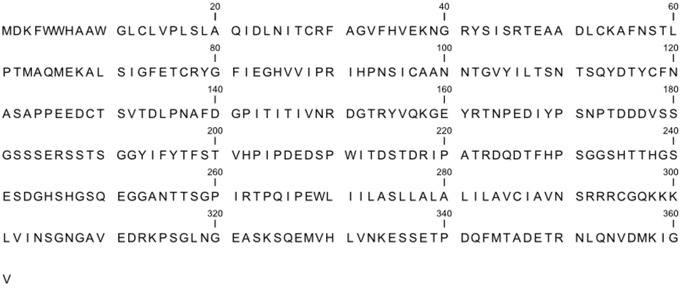	
Peptide Name	Sequence
CD44-1F	E^185^RSSTSGGYIFYTFFST
CD44-2	D^226^TFHPSGGSHTTHGSES^242^
CD44-2G	D^226^TFHPSGGS*HTT*HGSES
CD44-1GF	E^185^RSST*SGGYIFYTFFST
CD44-CS1	S^187^STSGGYIFYTFSTVHPIPDEDSPWITDSTDR^218^
CD44-3	G^252^GANTTSGPIRTPQIPE^268^
CD44-4	S^242^DGHSHGSQEGGANTTSGPI^261^
CD44-4G	S^242^DGHSHGSQEGGAN*TTSGPI^261^
CD44-1	E^185^RSSTSGGYIFYTFST^200^
CD44-1G	E^185^RSST*SGGYIFYTFST^200^
CD44-5	G^181^SSSERSSTS^190^

T* = Thr(α-D-GalNAc), S* = Ser(α-D-GalNAc), and N* = Asn(β-D-GalNAc).

It has been reported that highly efficient collagenolysis requires homodimerization of MT1-MMP [57]. The biologically relevant form of homodimerization is believed to be symmetrical, involving residues Asp385, Lys386, Thr412, and Tyr436 in blades II and III of the HPX domain [58], although other studies have suggested that homodimerization requires the outermost strand of blade IV [8]. However, homodimerization may be of weak affinity, and/or only a small fraction of cell surface MT1-MMP may be present as a dimer.

## 6. MT1-MMP as a Target in Cancer

Considering the major role of MTl-MMP in tumor invasion and metastasis [15,32], inhibition of its activity is an obvious therapeutic approach to block spreading of the cancer. With the recent recognition that MMPs can have protective roles in cancer, the concept of elevated expression of an MMP always being associated with worsened disease outcome is not always valid [59]. For example, primary breast cancer tumors in MT1-MMP-deficient mice developed faster than in their wild type counterparts, but showed a 50% reduction in metastasis [23]. Nonetheless, the correlation of MT1-MMP up-regulation with patients with metastatic breast cancer, and the inverse correlation of high MT1-MMP expression with patient survival time [8], strongly indicates application of selective MT1-MMP inhibitors in breast cancer. Correlation of MT1-MMP expression and malignant human brain tumors [60] indicates application of MT1-MMP inhibitors in this cancer type as well.

Ablation of the gene encoding MT1-MMP causes craniofacial dysmorphism, arthritis, osteopenia, dwarfism and fibrosis of soft tissues in mice [61]. This is the most significant phenotype among the MMP knockout mice, and suggests that the activity of MT1-MMP cannot be substituted by any of the other members of the family [1]. It also suggests that caution is needed in the development of MT1-MMP inhibitors. Further insight into the role of cell surface binding partners that modulate MT1-MMP activity may be needed for the development of new inhibitors.

## 7. Selective MT1-MMP Inhibitors

Attempts at developing selective MT1-MMP inhibitors have utilized small molecules, peptides, small proteins, and antibodies. Small molecule, hydroxamic acid-based inhibitors have been developed for MT1-MMP using a combinatorial library approach within subsites P_1_', P_2_', and P_3_' [62]. Although inhibitors with low nM IC_50_ values were obtained, selectivity between MT1-MMP and MMP-8 proved difficult [62]. Problems with hydroxamic acid-based metalloprotease inhibitors have been discussed in detail, particularly the tendency of hydroxamic acids to chelate zinc in a non-selective fashion [63]. An often observed side effect of hydroxamic acid-based MMP inhibitors has been musculoskeletal syndrome (MSS). MSS has been attributed to inhibition of MMP-1 and ADAM17/TACE [64]. A pyrimidine-2,4,6-trione derivative that inhibits MT1-MMP (as well as MMP-2 and MMP-9) is not associated with MSS, and thus demonstrates that better selectivity has the potential to create therapeutically useful MT1-MMP inhibitors [65].

Peptides spanning eight residues from the outermost strand of each MT1-MMP HPX domain blade were examined as inhibitors of MT1-MMP mediated cell migration [8]. Peptides IS4 (acetyl-Val-Met-Asp-Gly-Tyr-Pro-Met-Pro-NH_2_) and IVS4 (acetyl-Gly-Tyr-Pro-Lys-Ser-Ala-Leu-Arg-NH_2_) (administered at 100 μM concentration) were inhibitors. IS4 did not inhibit MMP-9 or MT6-MMP mediated COS cell migration, but did inhibit MT3-MMP mediated migration [8]. Mice bearing MDA-MB-435/GFP tumors were treated with 20 mg/kg peptide 6 day per week. The number and size of lung metastasis were significantly reduced by both peptides, with IVS4 being slightly more effective [8]. Peptide IS4 was subsequently found to directly inhibit MT1-MMP catalytic activity (Table 2).

**Table 2:** Inhibitors of MT1-MMP catalytic activities.

Inhibitor	IC_50_ (μM)	Reference
acetyl-Val-Met-Asp-Gly-Tyr-Pro-Met-Pro-NH_2_ (IS4)	3,400	[8,66]
Val-Phe-Asp-Glu-Ala-Ser-Leu-Glu-Pro-NH_2_	240	[66]
Gly-Ala-Cys-Phe-Ser-Ile-Ala-His-Glu-Cys-Gly-Ala (Peptide G)	150	[67]
N-TIMP-2	0.0008	[68]
N-TIMP-3	0.0008	[68]
DX-2400	~0.001-0.005	[69]

Peptide models of the 5 MT1-MMP HPX domain loops were synthesized and tested as inhibitors of MT1-MMP triple-helical peptidase activity [66]. Two of the peptides did not inhibit MMP-1 while being micromolar inhibitors of MT1-MMP. The best inhibitor was the peptide derived from MT1-MMP blade II strand 3-4, Val-Phe-Asp-Glu-Ala-Ser-Leu-Glu-Pro-NH_2_ (Table 2) [66].

The cyclic peptide G (Gly-Ala-Cys-Phe-Ser-Ile-Ala-His-Glu-Cys-Gly-Ala) was identified from phage display libraries as a selective MT1-MMP inhibitor (Table 2) [67]. Peptide G inhibited migration of HT1080 fibrosarcoma and C8161 melanoma cells on fibronectin. Treatment of mice injected with HSC-3 tumor cells with 2 mg/mL peptide G for 5 consecutive days reduced mean tumor volume by 54% and significantly enhanced survival [67].

N-TIMP-2 and N-TIMP-3 are very effective inhibitors of the MT1-MMP CAT domain (Table 2), but are not selective [68]. Overexpression of TIMP-2 significantly reduced melanoma growth in the skin of immunodeficient mice [70]. TIMP-1 has been mutated to create a more effective MT1-MMP inhibitor, but it is not selective [68,71,72].

A selective, fully human MT1-MMP inhibitory antibody (DX-2400; Table 2) has been developed using a human Fab displaying phage library [69,73]. DX-2400 was found to inhibit tumoral MT1-MMP activity, which in turn inhibited MDA-MB-231 primary tumor growth but not MCF-7 tumor growth in xenograft mouse models [69]. DX-2400 also inhibited metastasis [69].

Monoclonal antibody (mAb) 9E8 inhibits MT1-MMP activation of proMMP-2, but not other MT1-MMP catalytic activities [74]. Analysis of mAb 9E8 interaction with MT1-MMP determined that the antibody bound to the unique loop from Pro163 to Gln174 in the CAT domain [75]. Binding of mAb 9E8 to the loop interferes with TIMP-2 binding, preventing formation of the MT1-MMP•TIMP-2•proMMP-2 complex required for proMMP-2 activation [75]. Targeting of this loop has also proved effective for creating an MT1-MMP imaging agent (see below).

**Figure 4:**
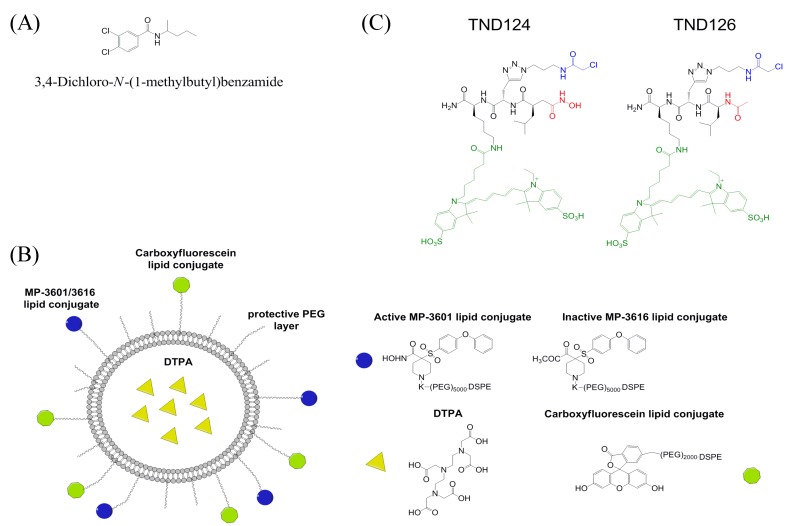
Structures of (**A**) MT1-MMP inhibitor NSC405020, 3,4-dichloro-*N*-(1-methylbutyl)benzamide, (**B**) MT1-MMP imaging agent MP-3653, and (**C**) MT1-MMP imaging agent TND124.

A small molecule MT1-MMP HPX domain inhibitor was identified using virtual ligand screening of the NCI/NIH Developmental Therapeutics Program ~275,000 compound library [76]. Compound NSC405020 [3,4-dichloro-N-(1-methylbutyl)benzamide] inhibited MT1-MMP homodimerization but not catalytic activity towards a fluorogenic peptide substrate or proMMP-2 activation (Figure 4A). NSC405020 was shown to reduce the collagenolytic activity of MCF7-β3/MT1-MMP cells. The compound was effective *in vivo*, as intratumoral injections reduced tumor size significantly.

As an alternative to active site inhibition, one my modulate MT1-MMP activities by affecting its subcellular localization. It has been observed that anti-tetraspanin antibodies affect MT1-MMP localization [6]. Since adult mice survive well without the tetraspanins CD9 and CD81 [6], approaches by which selective MT1-MMP-binding tetraspanins are targeted may have low toxicities.

## 8. Monitoring of MT1-MMP Activity

Visualization of membrane-bound, active MT1-MMP has been achieved by fluorescence resonance energy transfer (FRET) imaging of surface-anchored sensors. An initial MT1-MMP sensor was created using the Cys-Pro-Lys-Glu-Ser-Cys-Asn-Leu-Phe-Val-Leu-Lys-Asp sequence, derived from the MT1-MMP cleavage site in proMMP-2 [77]. The FRET pair was an enhanced cyan fluorescence protein (ECFP, the fluorophore) and a yellow fluorescence protein variant (YPet, the quencher) [77]. This indicator was anchored to the cell membrane by platelet-derived growth factor receptor transmembrane domain (PDGF_TM). A second generation sensor was created using the sequence Cys-Arg-Pro-Ala-His-Leu-Arg-Asp-Ser-Gly and the FRET pair mOrange2 (fluorophore) and mCherry (quencher) [4]. The MT1-MMP cleavage sequence was not a substrate for MMP-2 or MMP-9 [4]. MT1-MMP activity could be detected in HeLa cells to increase slowly and at the cell periphery upon epidermal growth factor stimulation [4]. However, the broad specificity of these sensors, based on short linear peptides, is unknown, and the ability to quantify MT1-MMP activity was not tested. Additionally, the change in fluorescence was not robust.

To improve selectivity compared with prior MT1-MMP probes, a pentapeptide library was screened, and the substrate C-10.1-Y (Ser-Leu-Ala-Pro-Leu-Gly-Leu-Gln-Arg-Arg) was found to be more selective for MT1-MMP compared with other MMPs, with the exception of MMP-9 [78]. MT1-MMP deficient breast carcinoma MCF-7 and MT1-MMP producing fibrosarcoma HT-1080 cells were screened with CyPet-[C-10.1-Y]-YPet, where CyPet was the fluorophore and YPet was the quencher, and compared with other MT1-MMP sequences. While all indicators had low backgrounds for the MCF-7 cells, CyPet-[C-10.1-Y]-YPet showed the greatest levels of MT1-MMP activity for HT-1080 cells.

A further optimized MT-MMP probe was developed based on the sequence AHLR (Cys-Arg-Pro-Ala-His-Leu-Arg-Asp-Ser-Gly) with Gly-Gly-Ser-Gly-Gly-Thr linkers flanking each side of the sequence [79]. The probe utilized the FRET pair ECFP (as fluorophore) and YPet (as quencher) [79]. MT1-MMP activity was favored over MMP-2, MMP-9, MT2-MMP, and MT3-MMP. Higher levels of MT1-MMP activity were detected in invasive breast cancer cells compared with non-invasive or benign cells. Adhesion of MDA-MB-231 cells to the fibronectin stimulated MT1-MMP activity. Simultaneous application of the probe with rhodamine-labeled collagen revealed MT1-MMP activity and collagen degradation in MDA-MB-231 cells [79].

Synthetic triple-helical peptide (THP) substrates have been developed for convenient, continuous assays of collagenolytic MMPs. The triple-helical FRET substrate fTHP-9 [(Gly-Pro-Hyp)_5_-Gly-Pro-Lys(Mca)-Gly-Pro-Gln-Gly~Cys(Mob)-Arg-Gly-Gln-Lys(Dnp)-Gly-Val-Arg-(Gly-Pro-Hyp)_5_-NH_2_, where (7-methoxycoumarin-4-yl)-acetyl (Mca) was the fluorophore and 2,4-dinitrophenyl (Dnp) was the quencher] is preferred by MT1-MMP compared with MMP-1 [80,81]. Our laboratory has quantified the triple-helical peptidase activity of MT1-MMP at the cell surface using fTHP-9 [82].

WT-MT1-MMP (wild type), MT1-MMP(ΔCT) (enzyme with the cytoplasmic tail deleted), MT1-MMP(ΔHPX) (enzyme with the HPX-like domain deleted), and MT1-MMP(MMP-1 CAT) (enzyme with the CAT domain of MMP-1) were stably transfected in MCF-7 cells. K_M_ for soluble MT1-MMP was 18.6 ± 1.4 μM and 15.1 ± 2.2 μM for WT-MT1-MMP, whereas k_cat_/K_M_ for the soluble enzyme was 3- to 4-fold higher as compared to the membrane-tethered enzyme (Table 3) [82]. Thus, the catalytic efficiency of the enzyme for fTHP-9 was less in the cell surface environment. It was reported previously that MT1-MMP differs in activity when in soluble form compared to surface-bound [40,57,83].

**Table 3:** Kinetic parameters for substrate hydrolysis by soluble MT1-MMP.

Substrate	K_M_ (μM)	k_cat_ (s^−1^)	k_cat_/K_M_ (s^−1^M^−1^)	Reference
Type I collagen (@ 27 °C)	2.9	0.0020	690	[83]
fTHP-9 (@ 37 °C)	18.6	0.84	45,130	[82]

Another MT1-MMP fluorogenic probe was designed based on the substrate sequence Gly-Arg-Ile-Gly-Phe~Leu-Arg-Thr-Ala-Lys-Gly-Gly and labeled with Cy5.5 and BHQ3 quencher (MT-P) [84] in an effort to target the membrane-bound form of MT1-MMP. MT-P was tested against MMP-2 and MMP-9 as well as against MT1-, MT2-, and MT3-MMPs. The probe displayed moderate selectivity. *In vivo* evaluation of probe activity in mice bearing MDA-MB-435 xenografts indicated strong near infrared (NIR) activation in the MT1-MMP-positive tumor region. Although MT-P displayed good specificity in the tumors, non-specific activation and accumulation was also observed in the liver. This result suggests further optimization of the probe is needed.

The non-substrate peptide His-Trp-Lys-His-Leu-His-Asn-Thr-Lys-Thr-Phe-Leu (denoted as MT1-AF7p), which displayed high binding affinity to the MT-loop region of MT1-MMP, was used to design and MT1-MMP NIR probe [85]. The MT-loop region is an eight amino acid insertion located within the CAT domain of MT-MMPs (MT1-, 2-, 3-, and 5-MMP). This insert is absent from all other MMPs. MT1-AF7 displayed the high affinity towards MT1-160p (^160^Arg-Glu-Val-Pro-Tyr-Ala-Tyr-Ile-Arg-Glu-Gly-His-Glu-Lys-Gln^174^) (K_d_ = 0.075 nM) [85]. MT1-AF7 was labeled with Cy5.5 (to create Cy5.5-MT1-AF7p) and evaluated in mice carrying MDA-MB-435 breast cancer xenografts (expressing high levels of MT1-MMP) and A549 xenografts (low MT1-MMP levels). Higher signal accumulation and better tumor contrast was observed in MDA-MB-435 xenografts.

In order to visualize trafficking of MT1-MMP, MT1-MMP-mCherry fluorescent construct was transfected and monitored with confocal time-lapse microscopy [86]. Interestingly, MT1-MMP-mCherry was shown to colocalize to vesicles localized to microtubules, and the coexpression of GFP-α-tubulin showed that the vesicles traveled in bidirectional fashion.

Another probe developed for MT1-MMP trafficking was a rhodamine X-conjugated anti-MT1-MMP antibody (anti-MT1-MMP mAb-ROX) [87]. Anti-MT1-MMP mAb-ROX was an activatable probe, with a quenched form containing approximately three ROX molecules per antibody. It was hypothesized that MT1-MMP internalization and delivery to lysosomes for degradation would activate the fluorescent probe. The probe was validated in C6 glioma cells and MCF-7 breast cancer cells as MT1-MMP positive and negative models, respectively. The intake of anti-MT1-MMP mAb-ROX bound to MT1-MMP was measured and compared to cells treated with nonspecific ROX-labeled antibodies (NC ab-ROX). The fluorescence intensity was significantly higher in C6 cells compared to MCF-7 cells, and when anti-MT1-MMP mAb-ROX was used compared with NC ab-ROX. These effects could be negated by blocking endocytosis. The probe was successfully tested in other cell lines as well (MDA-MB-231 and HT-1080). The probe was tested *in vivo* in C6 and MCF-7 xenografted mice and displayed highly differential tumor to muscle (T/M) and tumor to basal (T/B) signal in C6-implanted mice and not in MCF-7 mice. The T/M ratios increased ~10-fold within 24 h of injection, and 15-fold within 48 h, as compared to 2.6-fold and 4.6-fold, respectively, for NC ab-ROX. The advantage of this system is that the anti-MT1-MMP antibody had high specificity and affinity for its target, thus providing strict imaging of MT1-MMP. However, the fluorescence of the probe was too short for the use for *in vivo* imaging and the system may be improved by replacing ROX with a tissue-penetrating NIR group.

In order to evaluate MT1-MMP activity in MDA-MB-231 cells, a tripartite probe was designed containing the following components: (i) a positively charged D-Arg octamer (r8) cell penetrating peptide (CPP) attached with single amino acid chelate (SAAC) for technetium-99m; (ii) a MT1-MMP specific substrate (Ser-Gly-Arg-Ile-Gly-Phe-Leu-Arg-Thr-Ala); and (iii) a negatively charged D-Glu attenuation sequence [7]. Several attenuation sequences were evaluated in order to achieve linear conformation of the cleavable sequence that would be available for MT1-MMP docking. The sequence comprising of four D-Glu-Gly-Gly repeats (4egg) was chosen for cell-based studies. Probe activation was determined by treatment of transfected MDA-MB-231 cells with and without a broad spectrum MMP inhibitor (GM1489). The average uptake of the 4egg probe was two times greater in untreated cells indicating successful cleavage and increased uptake of the activated probe into MT1-MMP expressing cells. Negative results from cells treated with free ^99m^Tc-tricarbonyl complex ([99mTc(CO)3]^+^) suggested that there was no non-specific uptake of the radiolabel and that the ^99m^Tc accumulation in cells treated with the intact probe was the result of cleavage rather than leakage of free radiolabel into these cells. Further *in vivo* imaging analysis will be needed to determine clinical potential of this probe.

Antibody radiolabeling has also been explored as a potential novel probe for MT1-MMP activity [88]. ^99m^Tc-anti-MT1-MMP monoclonal antibody (mAb) was evaluated in breast tumor-bearing rodents. MT1-MMP was highly expressed in all malignant cells tested. Tumor radioactivity increased over time and displayed 3 to 5-fold increase at 24 h as compared to 1 h after antibody injection. The radio-antibody cleared other organs and blood rapidly suggesting a promising *in vivo* application of this probe.

MT1-MMP imaging on the cell surface has been achieved using carboxyfluorescein, where the imaging agent is hydroxamate-based with low nM affinity for active MT1-MMP (Figure 4B) [89]. MP-3653 has *N*-hydroxy-4-((4-phenoxyphenyl)sulfonyl)piperidine-4-carboxamide-PEG(5000)-DSPE (the hydroxamate, MP-3601) and DSPE-PEG(2000)-carboxyfluorescein inserted into liposomes (hydrogenated soybean PC, cholesterol, DSPE-PEG(2000), 11:8:1 molar ratio). MP-3653 was used to visualize trafficking of MT1-MMP through cell compartments, and to quantify the number of active MT1-MMP molecules per cell [89]. However, the imaging agent is not selective between MT1-MMP, MMP-2, and MMP-9 [89].

MT1-MMP was mutated at position 260 to introduce a Cys in place of the wild-type Phe [90]. This mutation is near the active site of MT1-MMP but has little effect on enzyme activity [90]. The Cys was then used for attachment of a specific probe, TND124, which contained a hydroxamate (for targeting the active site zinc), an electrophile (α-chloro acetamide, for reacting with the Cys residue), and a fluorophore (Cy5, for imaging) (Figure 4C) [90]. The probe had an IC_50_ value of 1 μM for the mutant enzyme, and was used to image the mutant enzyme in HEK293T cells and in zebrafish [90]. One could introduce the mutant enzyme into mice and monitor the role of MT1-MMP in rodent cancer models.

## 9. Conclusions

Through a vast number of studies, MT1-MMP has been recognized as having a critical role in cancer progression. Given the great variety of MT1-MMP substrates, as well as non-catalytic MT1-MMP functions, it is a daunting task to identify the precise mechanisms by which MT1-MMP facilitates cancer progression. Selective MT1-MMP inhibitors, specifically those that target discreet functions of MT1-MMP, hold great promise for anti-MT1-MMP therapies. These inhibitors could modulate the disease-promoting aspects of MT1-MMP while allowing physiologically favorable MT1-MMP activities to proceed unabated. The monitoring of MT1-MMP *in vivo* can be used to diagnose the effectiveness of selective inhibitors as anti-cancer agents.
